# Influence of alumina shot blasting induced roughness on bacterial adhesion to titanium

**DOI:** 10.1007/s00784-025-06580-2

**Published:** 2025-10-11

**Authors:** Marta Romero-Serrano, Manuel María Romero-Ruiz, José Vicente Ríos-Santos, Blanca Ríos-Carrasco, Javier Gil

**Affiliations:** 1 Periodontics Department, Faculty of Dentistry. University of Seville, c/Avicena s/n, Seville, 41009 Spain; 2 Bioinspired Oral Biomaterials and Interfaces. Materials Science and Engineering Department. EEBE, Technical University of Catalonia, Eduard Maristany Av. 16, Barcelona, 08019 Spain

**Keywords:** Roughness, Bacteria, Shot blasting, Alumina, Wettability, Residual stress

## Abstract

**Objective:**

To evaluate the influence of different surface roughness levels of titanium disks, induced by alumina blasting, on bacterial adhesion.

**Materials and Methods:**

Twelve different surface roughnesses, ranging from 0.01 μm to 6 μm, were produced using a shot blasting technique with varying alumina particle sizes. Surface roughness was measured using confocal interferometry, wettability was assessed by contact angle measurements, and compressive residual stress was evaluated by X-ray diffraction. For each roughness level, 720 samples were used to culture Porphyromonas gingivalis (Gram-negative, anaerobic) and Streptococcus sanguinis (Gram-positive, anaerobic). The colonies formed per unit area, the ratio of dead bacteria to total bacteria, and the metabolic activity for each roughness ere determined.

**Results:**

The polished surface (Sa = 0.01 μm) showed the highest bacterial adhesion for both strains compared to the 0.13 μm roughness, which exhibited a antibacterial activity, likely due to nanostructured peaks causing bacterial membrane disruption. For surface roughness values between 0.5 and 3 μm, Gram-positive bacterial colonies increased approximately threefold. When the roughness exceeded 3.8 μm, colony formation rose fivefold. In contrast, Gram-negative bacteria did not exhibit statistically significant changes in adhesion between 0.5 and 2 μm. However, beginning at 2.6 μm, a marked increase was observed, with colony numbers reaching nearly four times the control at 6 μm. The ratio of dead bacteria and metabolic activity confirms bacterial colonization studies (CFU/mm^2^).

**Conclusions:**

Surface roughness significantly influenced bacterial colonization on titanium implants. An antibacterial effect was observed at a roughness of 0.13 μm. Bacterial adhesion increased moderately up to 2.1 μm for Gram-negative and 3 μm for Gram-positive strains, followed by a sharp rise at higher roughness values. An optimal surface roughness range of 1 to 2 μm appears to promote favorable osteoblastic response while minimizing bacterial adhesion.

**Clinical Relevance:**

These results enhance our understanding of how implant surface roughness influences bacterial adhesion. This knowledge could contribute to the development of clinical approaches designed to lower the risk of peri-implantitis

**Supplementary Information:**

The online version contains supplementary material available at 10.1007/s00784-025-06580-2.

## Introduction

Dental implants have revolutionized modern dentistry by providing a reliable and long-term solution for tooth replacement. Despite their success, one of the major challenges associated with dental implants is the risk of bacterial colonization, which can lead to peri-implant diseases such as peri-implant mucositis and peri-implantitis [1, 2]. These infections can compromise implant stability and longevity, ultimately leading to implant failure. Among the various factors influencing bacterial adhesion, surface roughness plays a crucial role. The roughness of an implant surface can affect both the biological integration of the implant into the bone (osseointegration) and its susceptibility to bacterial colonization [3, 4]. This report aims to explore in detail the relationship between surface roughness and bacterial adhesion, examining the mechanisms involved, the findings of experimental studies, and potential strategies to mitigate the risks associated with bacterial colonization.

The roughness of dental implants is generally classified into three main categories: *Smooth surfaces (Sa < 0.2 μm)*, offering minimal roughness, which reduces bacterial adhesion but also hinders optimal osseointegration; *moderately rough surfaces (Sa between 0.2 and 2 μm)*, which strike a balance between promoting osseointegration and minimizing bacterial adhesion, and h*ighly rough surfaces (Sa > 2 μm)*, which enhance osteoblastic activity, but also significantly increase the risk of bacterial colonization by facilitating the presence of micro-irregularities where bacteria accumulate and grow [5, 6].

Bacterial adhesion is a crucial step in the formation of biofilms, which allows them to establish persistent colonies on a wide range of surfaces, providing them with increased resistance to antimicrobial treatments, making it difficult to eradicate infections. Given the clinical and industrial importance of bacterial adhesion, numerous investigations have been carried out to elucidate its underlying mechanisms and develop effective strategies to control biofilm-associated infections [7–9].

Before adhering, bacteria must approach the surface, which they achieve through diffusion, fluid dynamics and the bacteria’s own activity. Upon approach, an attraction occurs due to van der Waals forces (Fa), followed by a repulsive force due to the negative charge of the bacteria (Fe); the interaction between these two forces will determine the final distance the bacteria will approach (Gibbs free energy) [10].

Biofilms provide bacteria with a protective environment that increases their resistance to antimicrobial agents and host defenses, and may even release free-floating bacterial cells, contributing to the progression of infection. The relationship between surface roughness and bacterial colonization is complex, as varying degrees of roughness can influence bacterial attachment, biofilm formation, and peri-implant disease progression in different ways. Research has shown that bacterial colonization increases with surface roughness due to the creation of more attachment points and microspaces in which bacteria can proliferate [11, 12]. So, highly rough surfaces (Sa > 2 μm), tend to accumulate greater numbers of bacteria due to their increased surface area and micro-irregularities. These irregularities create niches where bacteria, through the formation of biofilms, can attach, proliferate and evade host immune responses, antimicrobial treatments and mechanical cleaning. On the other hand, smoother surfaces (Sa < 0.2 μm) exhibit significantly reduced bacterial adhesion. However, excessively smooth surfaces may negatively impact osseointegration, as osteoblast adhesion and bone formation are also influenced by roughness. Thus, an optimal balance must be achieved, typically with moderately rough surfaces (Sa 0.2–2.0 μm), which promote osseointegration while minimizing bacterial colonization [13].

However, it should be noted that implant roughness is not the only factor influencing bacterial adhesion and biofilm formation and maturation. Different factors can contribute as well as surface roughness. These factors can be divided into four groups [14, 15]:

- Biomaterial-related properties; chemical composition, surface charge (zeta potential), hydrophobicity/hydrophilicity, surface contaminants….
- Oral and host-related factors; salivary proteins, patient oral hygiene, periodontal and peri-implant diseases and conditions, implant or prosthesis design, ….
- Microbiological aspects; type for colonizing bacteria, interbacterial interactions, extracellular polymeric substances production….
- Clinical and systemic conditions; detrimental occlusal load, different systemic factors as diabetes, smoking, immunosuppression….

The surrounding environment also significantly affects bacterial adhesion. Factors such as pH, ionic strength, temperature, and nutrient availability can alter bacterial behavior and adhesion dynamics; so, variations in environmental pH and ion concentration can influence bacterial surface charge and adhesion strength, and some bacteria even exhibit optimal adhesion at specific pH levels. It is known that elevated temperatures can enhance bacterial adhesion by increasing metabolic activity and promoting the expression of adhesion-related proteins. When nutrients are limited, bacteria may activate adhesion mechanisms to secure a stable environment for survival and proliferation [16–18].

Currently there are several bactericidal strategies depending on the typology of the main products used, thje different strategies used can be classified as follows:

- Topographical bactericides: Due to the characteristics of surfaces, we can find great differences in the adhesion and proliferation of bacteria on them. A clear example is roughness. There is a dilemma here because roughness favors bacterial adhesion but at the same time increases osseointegration, so a balance must be found between both actions [19].

The importance of nanotopography and surface topography in bacterial adhesion and biofilm formation has been recognized in last years. It is now known that bacteria can colonize surfaces with an average roughness (Sa) of just a few nanometers. Metal surfaces with nanometer or sub-nanometer scale roughness have been shown to resist or prevent bacterial attachment of certain types of bacteria, due to unfavorable surface topography [20].

- Chemical bactericides: Certain elements, such as antibiotics, are very effective against bacteria. Some of them can treat different types of very specific bacteria [21].
- Bactericides based on functionalization: the most important would be the silane group, whose functionalization is called silanization, in which silane molecules act as a binder between the substrate and biomolecules. They allow covalent binding to the surface of peptides and proteins, which has an effect on bacteria. Some silanes can induce the same effects on their own [22, 23].

Another aspect to consider is the effect of the biomaterial surface topography in the transepithelial zone on biofilm formation, especially in the supragingival areas, as an entry route for germs that can subsequently spread to subgingival areas and then to the implant surface. Studies have shown on different restorative materials that transmucosal implant surfaces with a higher surface roughness facilitate biofilm formation [24].

The objective of this study is to determine the influence, over a wide range of roughness, of the behavior of two of the most important bacteria in the formation of biofilm that generates peri-implantitis disease. The originality and interest of this research is that all parameters that could affect bacterial colonization, such as wettability, surface retained alumina, compressive residual stress remain constant in the microbiological tests so that only roughness is the variable that affects bacterial growth. This behavior will allow us to obtain the best topographical compromise for poor bacterial colonization.

## Materials and methods

### Titanium discs

We performed a study of 12 different roughness from 0.01 (polished) to 6 μm, using commercially pure titanium grade 3 discs (Cp-Ti) that were treated by shot blasting method with different sizes of alumina particles. An incidental non-probabilistic finite sample of 876 titanium discs of each roughness for different physical-chemical surface characterization and the microbiological studies for each bacterial strain were kindly provided by SOADCO S.L. (Les Escaldes Engordany, Andorra). Discs used in this study with dimensions of 5 mm in diameter and 2.5 mm thick.

An MPA 600 shot blasting machine was used, which projects by means of air flow at variable pressure. Alumina abrasive particles of different sizes from 0.4 to 10 μm were used. To obtain the smaller particles, 1 μm particles were placed in an agate planetary ball mill to grind the particles to a particle size in the range of 0.2 to 0.6 μm. The distance from the gun to the titanium surface was 150 mm and the air pressure was 2.0 bar.

### Roughness

Surface roughness refers to the microscopic topographical characteristics of a material’s surface and is quantified using various parameters, the most common being Sa (Arithmetic mean height, a 3D measurement; Sa quantifies surface roughness by measuring the average height difference between the surface’s peaks and valleys), Sy (Maximum height; represent the highest point), St (Total height; sum of the maximum and minimum heights, reflecting the overall height variation), Sm (Mean crest height; quantifies the average height of the surface peaks. It provides information about the overall crest height characteristics of the surface) and Pc values (Peak curvature; curvature of the surface peaks. They are useful for understanding the shape and sharpness of the surface features [5].

The surface roughness measurements were done with a white light interferometer microscopy (Optical Profiling System, Wyko NT1100, Veeco, USA). This method, unlike the profilometer, has a 3-dimensional image capture and allows to have volumetric values with resolutions of 100 nm sufficient for this research. Another method could be the use of Atomic Force Microscopy but for large roughness it does not have the adequate resolution in relation to interferometric microscopy.

Three samples of each of the different shot-blasting and sterilization series were analyzed and for every sample three readings of rugosity were carried out.

It is well known that the geometric structure of rough surfaces is random, and that roughness features are found at a large number of length scales between the length of the sample and atomic scales. Where nanometer surface resolution was required, optical interference techniques were employed. These systems work on the principle of interference of two beams of light where at least one is reflected off the surface of the specimen [16].

A phase-correct filter, a Gaussian cut-off filter, was used to separate the waviness and form from the roughness. In our case, it was used a cut-off value (λc) of 2.5 for roughened samples which covers 2.0 < Sa range ≤ 10.0 and λ_c_ = 0.25 for smoothed samples.

Several roughness parameters exist to describe surface topography: amplitude, spacing and hybrid roughness parameters. In this study, the following parameters were used: (1) Amplitude parameters: these are only high descriptive, for example Sa, Sm, Sq, and Sm [6]. (2) Spacing parameters: these describe the spacing between the topographical irregularities, for example Pc. The index area was also considered. None hybrid parameters were studied since any relevant information was obtained.

### Wettability

The measurement of the static contact angle was carried out through the sessile drop method because the angle of contact is constant over time. The number of discs analyzed was 5 for each roughness. Drops were generated with a micrometric syringe and deposited on the substrate surface, inside a chamber saturated with the liquid under study at 37 °C. The wettability studies were obtained with a contact angle video-based system (Contact Angle System OCA15plus, Dataphysics, Germany) and analyzed by the SCA20 software (Dataphysics, Alemania). At the rough surfaces, droplets confront two different conditions. In the first mode, the water droplet contacts with the rough surface placed under the drop, resulting in wetting of all the grooves below the drop surface, Wenzel explained that at the rough surface, the actual area of the solid–liquid contact under the drop was greater than the flat surface [25]. Modified Wenzel’s Eq. (1) is:

1 $$cos\theta W\:=\:rcos\theta Y$$

where θ_W_ is the apparent contact angle in Wenzel’s theory and r is the surface roughness ratio, which is obtained in accordance with Eq. (2). In fact, r is the ratio of the actual surface, Ar, to the geometric surface, A_0_ [26, 27]:

2 $$r\:=\:ArA0=cos\theta Wcos\theta Y$$

### Residual stress

Residual stresses were measured with a diffractometer incorporating a Bragg-Bentano configuration (D500, Siemens, Germany) for 5 samples for each roughness. The measurements were done for the family of planes (213), which diffracts at 2θ = 139,5^o^. The elastic constants of Ti at the direction of this family of planes are EC = (E/1 + n) _(213)_ = 90,3 (1,4) GPa. Eleven Ψ angles, 0^o^ and five positive- and five negative-angles were evaluated. The position of the peaks was adjusted with a pseudo-Voigt function using appropriate software (WinplotR, free access on-line), and then converted to interplanar distances (dΨ) using Bragg’s equation. The d Ψ vs. sen2Ψ graphs and the calculation of the slope of the linear regression (A) were done with appropriate software (Origin, Microcal, USA). The residual stress is: σ = EC(1/d_0_) A; where d_0_ is the interplanar distance for Ψ = 0^o^.

### Microbiological study

Two bacterial strains were used in the study; *Porphyromomas gingivalis*, an anaerobic Gram - bacteria, one of the most abundant biofilms in peri-implantitis, and *Streptrococcus sanguinis*, an anaerobic Gram + bacterium that plays an important role in biofilm formation. A total of twelve different titanium surfaces were studied with different roughness and each bacteria strain. Standard reference strains of *Streptococcus sanguinis*, (ATCC 6249), *Porphyromonas gingivalis (*ATCC 33277) were purchased from Microbiologics (MN, USA) and used for the cultures. We used 30 titanium discs with 5 mm in diameter of each surface and each bacteria strain. Three different types of microbiological characterization were realized: Determination of colonies formation unit, live and dead test with the determination of the ratio of bacterial dead and metabolic activity test by Resazurin reduction.

The total discs used were:

$$\begin{array}{l}\left(20forCFUs\:+\:5forlive/deadtest\:+\:5formetabolicactivity\right)\\\times12\:\times\:2\:=\:720discs\end{array}$$

#### CFU determination

480 discs were washed with 70% ethanol, acetone and distilled water, dried at room temperature and sterilized in autoclave. The quantification of bacteria attachment was done with two normal inhabitants of the mouth: Cp-Ti discs was incubated with bacteria broth (specific for each strain) for 2 h at 37 °C and 5% CO_2_, washed with physiological body simulation (PBS) and detached in Ringer’s solution. New bacteria suspension was seeded on MRS solid medium for *Porphyromonas gingivalis*, and Todd-Hewitt for Streptococcus sanguinis), incubated for 48 h at 37 °C and finally the number of colonies were counted [28–30]. At the same time the pH variation during bacteria growth phase was measured using SevenCompact Duo pH/Conductivity pH meter (Mettler Toledo, Mumbai, India). The count was performed manually using a Zeiss Axiolab 5 microscope.

#### Live/dead and ratio of dead bacteria determination

120 discs were sterilized with 70% ethanol for 5 min, washed twice with PBS, and incubated with 1 ml of bacterial suspension of Porphyromonas gingivalis or Streptococcus sanguinis for 4 h at 37 °C. Afterwards, the supernatant was removed, samples were washed with PBS and incubated with a solution containing LIVE/DEAD BackLight Bacterial Viability Kit (ThermoFisher, US) at room temperature in the dark for 15 min. A Zeiss LSM 800 confocal microscope (Carl Zeiss, Germany) was used to observe the samples with a 63 lens. The images of the SYTO-9 and Propidium iodide (PI) staining were acquired using Zen 2.3 software (Carl Zeiss). The number of bacteria stained with SYTO-9 was used to calculate the percentage of bacterial adhesion to the samples. The adhesion values were normalized in reference to the SB condition values. All the assays were performed in triplicate using three samples of each condition.

The confocal LIVE/DEAD images were analysed and quantified using ImageJ software. The ratio of dead bacteria of red fluorescence (damaged cells) versus total cells (sum of green and red) indicated the portion of dead or compromised cells for each treatment. All the assays were performed in triplicated using three samples per each condition.

$$\:ratio\:of\:dead\:bacteria=\frac{red\:bacteria}{greeen\:bacteria+red\:bacteria}$$

#### Metabolic activity

For the metabolic assay, 120 discs and the positive controls were incubated with 650 µL of 25 µg/mL resazurin sodium salt in PBS (Sigma-Aldrich, St. Louis. MO, USA) at 37 °C until the positive control was saturated. Then, 100 µL was used to read the absorbance at 570 and 600 nm, and this was used to calculate the reduction percentage. Resazurin reduction is a metabolic test that uses the blue, non-fluorescent dye resazurin to detect metabolically active and viable bacterias. Bacteria reduce resazurin into a red, highly fluorescent product called resorufin through respiration or other enzymatic processes. This color and fluorescence change from blue to pink/red can be measured to quantify bacteria viability. The measurement was realized by Confocal Laser Scanning Microscope Zeiss LSM 800.

### Statistical analysis

Data are expressed as means and standard error of the mean (SE), and their normality was assessed using the Shapiro–Wilk test. A general linear model was developed to compare the bacterial counts for each bacteria strain. To compare the live/dead ratios from the CLSM data, a one-way ANOVA with Bonferroni’s corrections for multiple comparisons was applied. Results were considered statistically significant at *p* < 0.05. All statistical analyses were performed using a software package (IBM SPSS Statistics 29.0; IBM Corporation, Armonk, NY, USA).

Number total of discs used: (3 discs for determination of roughness + 5 discs for wettability characterization + 5 for determination of compressive residual stress) x 12 roughness studied + (20 discs for CFUs + 5 discs for live/dead test + 5 discs for metabolic activity) x 12 roughnesses studied x 2 bacteria strains = 876 discs.

## Results

**Fig. 1:**
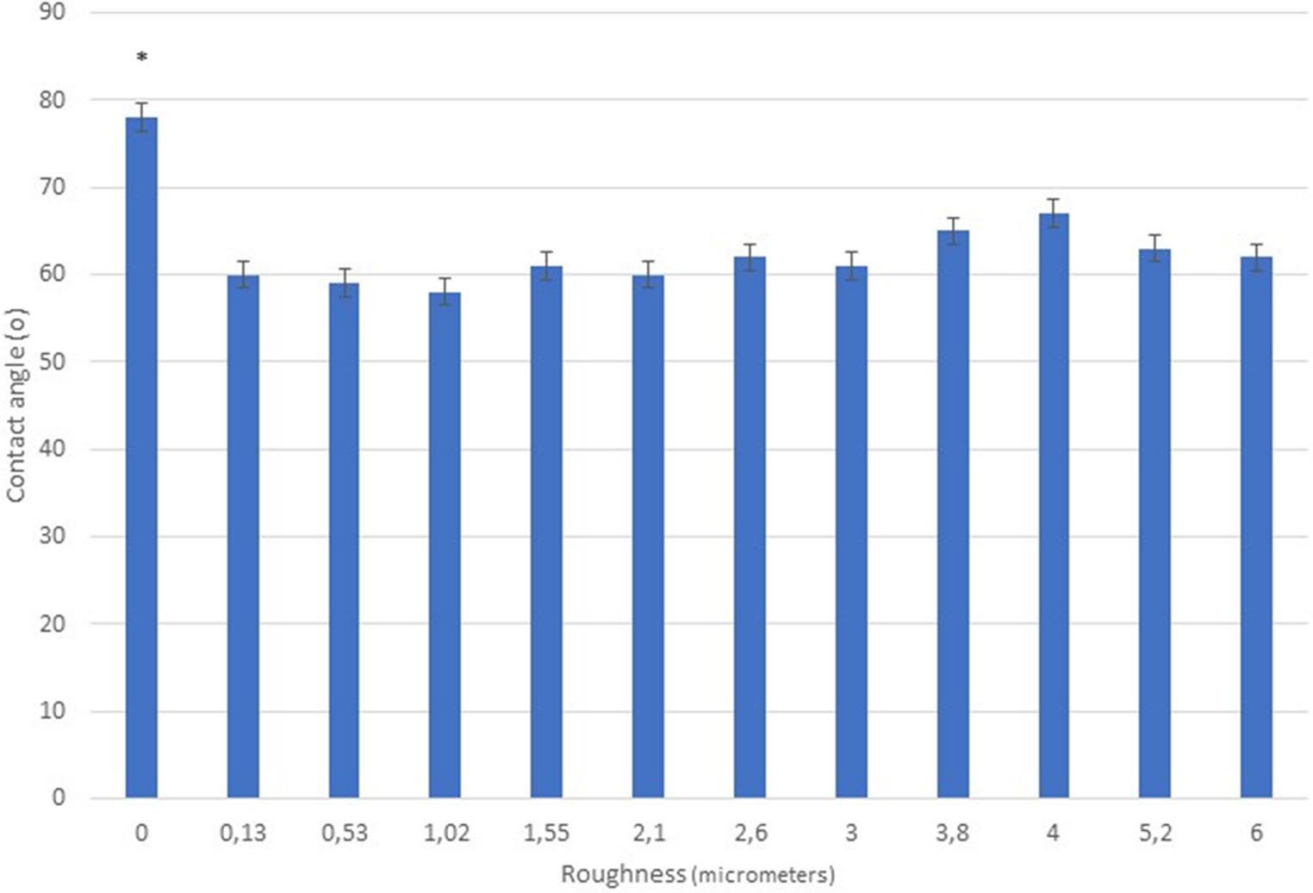
Contact angle corrected by Wenzel equation for the different surfaces studied. The bars are the standard error of the mean. The asterisk indicates the statistical differences significance with *p* < 0.05. The p-values for each condition are shown in the Supplement. (*n* = 5 samples for each roughness with 3 measurements by sample)

Table 1 shows the roughness parameters obtained for the different shot blasting treatments with alumina particles. Figure 1 shows the contact angles corrected by the Wenzel equation for the different roughness Sa. No statistically significant difference of wettability with roughness is observed.

**Table 1:** Roughness parameters for the different treatment of the cp. titanium discs

	Sa (µm) ± SEM	Sy (µm) ± SEM	Sm (µm) ± SEM	Pc (1/µm) ± SEM	Index Area ± SEM
Polished	0.01 ± 0.03	0.23 ± 0.01	9.48 ± 0.34	43.39 ± 6.24	1.04 ± 0.01
1	0.13 ± 0.01	0.35 ± 0.02	10.00 ± 0.26	35.37 ± 4.21	1.02 ± 0.01
2	0.53 ± 0.03	0.87 ± 0.04	12.25 ± 1.11	23.30 ± 3.47	1.07 ± 0.01
3	1.02 ± 0.03	1.89 ± 0.22	15.00 ± 1.33	13.90 ± 1.81	1.10 ± 0.01
4	1.55 ± 0.03	5.56 ± 0.37	24.33 ± 1.18	10.55 ± 1.01	1.09 ± 0.01
5	2.10 ± 0.03	8.32 ± 0.69	30.12 ± 2.22	7.89 ± 0.44	1.17 ± 0.01
6	2.60 ± 0.03	12.32 ± 0.34	35.23 ± 1.78	5.18 ± 0.18	1.20 ± 0.01
7	3.00 ± 0.03	18.29 ± 0.16	42.13 ± 0.85	6.79 ± 0.15	1.27 ± 0.02
8	3.80 ± 0.08	26.89 ± 0.46	53.29 ± 0.90	5.21 ± 0.20	1.42 ± 0.03
9	4.00 ± 0.02	16.63 ± 0.21	43.72 ± 0.23	8.41 ± 0.10	1.30 ± 0.03
10	5.20 ± 0.08	25.46 ± 1.71	52.84 ± 1.07	5.18 ± 0.20	1.37 ± 0.03
11	6.03 ± 0.12	32.33 ± 0.57	60.23 ± 3.62	4.32 ± 1.01	1.57 ± 0.03

Sa (Arithmetic mean height), sy (Maximun height), Sm (Mean crest height), Pc (Peak curvature) and index area as the ratio of the actual surface area of the sample to the ideal surface area if there were no roughness. SEM is the standard error of the mean. (n = 3 samples with 3 measurements by sample)

**Fig. 2:**
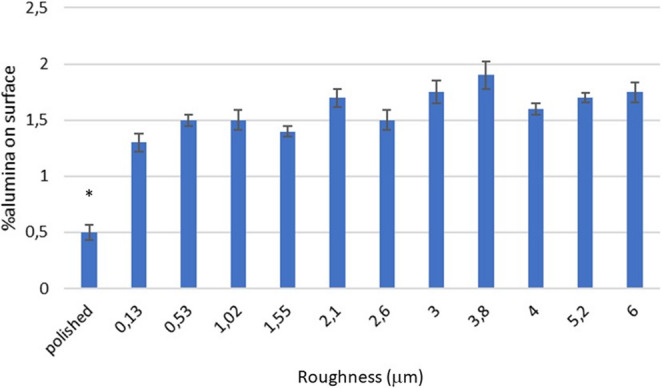
Percentage of residual alumina on the titanium surface for the different surfaces studied. The bars are standard error of the mean. The asterisk indicates the statistical differences significance with p < 0.05. The p-values for each condition are shown in the Supplement. (n = 5 samples for each roughness with 3 measurements by sample)

One of the parameters that could affect the physicochemical properties of the surface would be the amount of residual alumina remaining on the surface of the dental implant. The polar characteristics of the aluminum oxides could vary the wettability. However. we have been able to verify that the different shot blasting treatments leave alumina residues around 1.5% and between them there are no statistically significant differences and therefore this variable can be considered constant on titanium surfaces. The only significantly lower residual alumina value is in the case of polishing which by X-ray energy dispersive microanalysis values of 0.5% have been obtained. This residue must come from the polishing treatments that are made with suspensions of alumina particles and diamond paste. These results can be seen in Fig. 2.

**Fig. 3:**
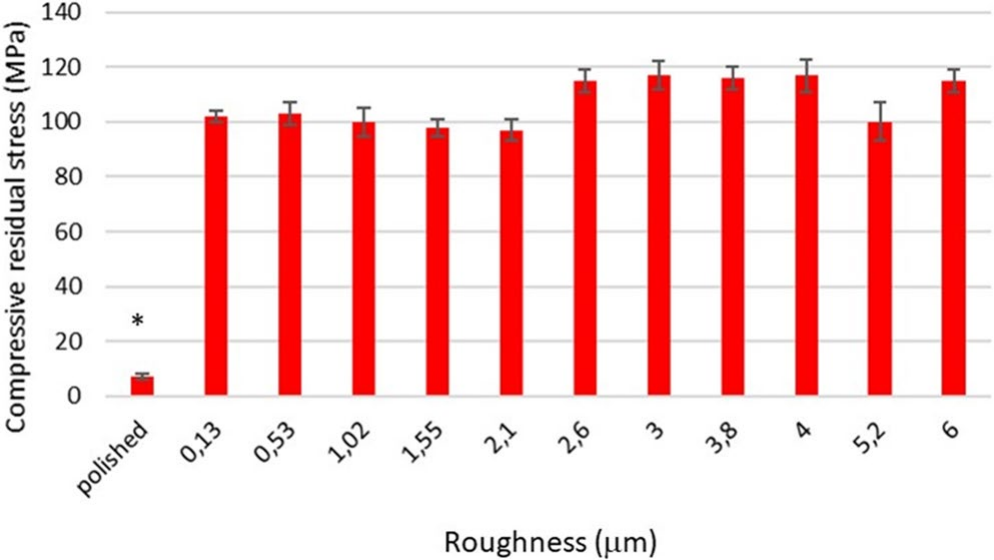
Compressive residual stress produced by the shot blasting process for each surface studied. The bars are standard error of the mean. The asterisk indicates the statistical differences significance with *p* < 0.05. The p-values for each condition are shown in the Supplement. (*n* = 5 samples for each roughness with 3 microanalyses by sample)

Figure 3 shows the compressive residual stress obtained by X-ray diffraction of the different treatments. It can be observed that the values are compressive around − 100 MPa and between them there are no statistically significant differences. However. the polished sample offers a lower compressive residual stress since in this case the titanium has not been treated by projection of abrasive particles but by polishing with cloths with alumina suspensions and diamond powder.

The results allow us to deduce that the residual stress depends on the spraying pressure. which in our case remained constant at 2.5 bar. Another factor that could affect the residual stresses would be the abrasiveness of the particles but also the alumina used differs only in the particle size.

**Fig. 4:**
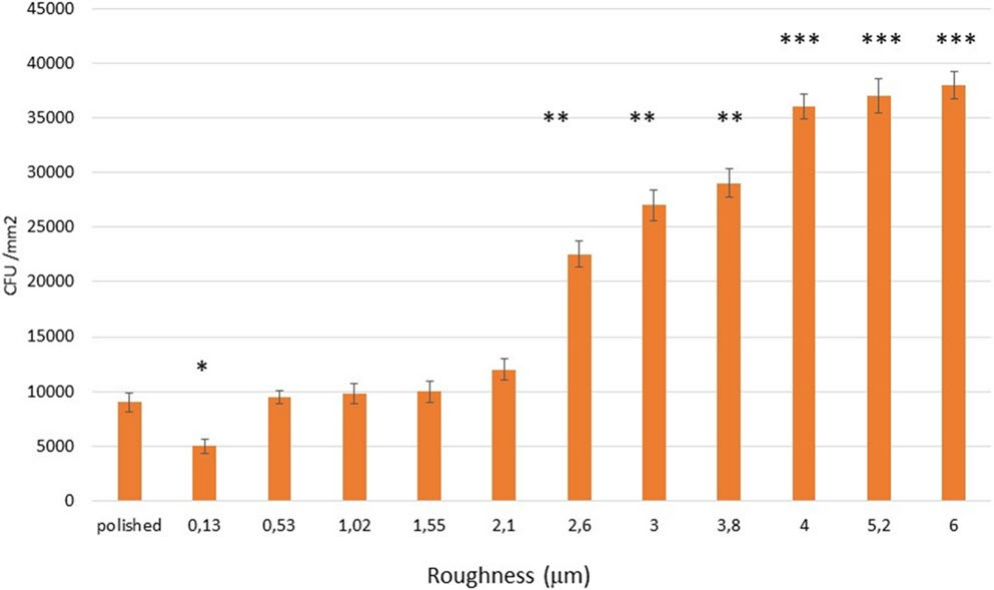
*Porphyromonas gingivalis colony* forming units per mm^2^ (CFU/mm^2^) for each surface studied. The asterisks indicate the statistical difference significance with *p* < 0.05. One asterisk is statistically different of two and three asterisks and two and three asterisks are different between them. The p-values for each condition are shown in the Supplement. The bars are standard error of the mean. (*n* = 20 discs for each roughness studied)

When performing the microbiological studies. the CFU/mm^2^ of the *Porphyromonas gingivalis* colonies (Gram -) can be observed in Fig. 4. where three remarkable situations can be clearly seen. Firstly. it can be seen that the samples with a roughness of 0.13 μm present statistically significant differences with the specularly polished surface with a Sa of 0.01 μm. At higher roughness. a slight increase is observed up to 2.1 μm Sa and thereafter a linear increase up to roughnesses around 4 μm. above which there is a sharp increase.

**Fig. 5:**
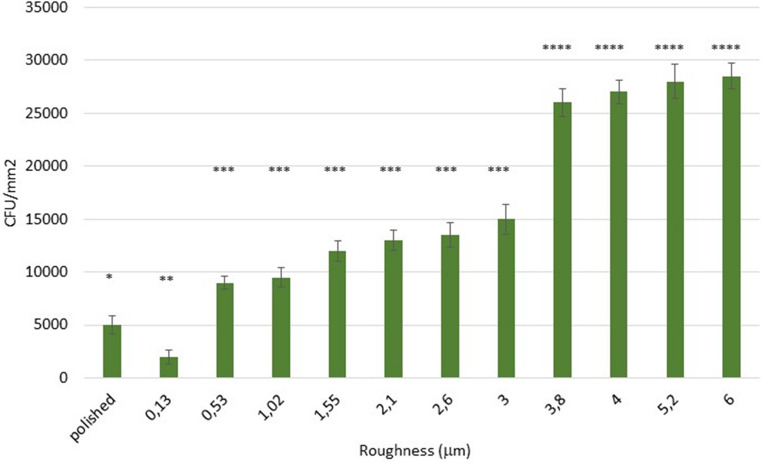
*Streptococcus sanguinis colony* forming units per mm^2^ (CFU/mm^2^) for each surface studied. The asterisks indicate the statistical difference significance with *p* < 0.05. One asterisk is statistically different of two and three asterisks and two and three asterisks are different between them. The same meaning is for four asterisks. The p-values for each condition are shown in the Supplement. The bars are standard error of the mean. (*n* = 20 discs for each roughness studied)

In the case of *Streptococcus sanguinis (*Gram +). a behavior quite similar to that of Gram + can be observed in Fig. 5. where the roughness of 0.13 is also lower than the polished one and from this roughness there is a growth of the bacterial colonization of low slope (between 0.53 and 3 μm). and from the roughness of 3.8 μm there is an abrupt increase as in the case of *Porphyromonas gingivalis*.

The pH values were monitored in the colony formation tests and no changes in the acidity of the suspension were observed, ensuring that the procedure was carried out correctly. The pH values obtained were slightly acidic, between 6.2 and 7.0.

**Fig. 6:**
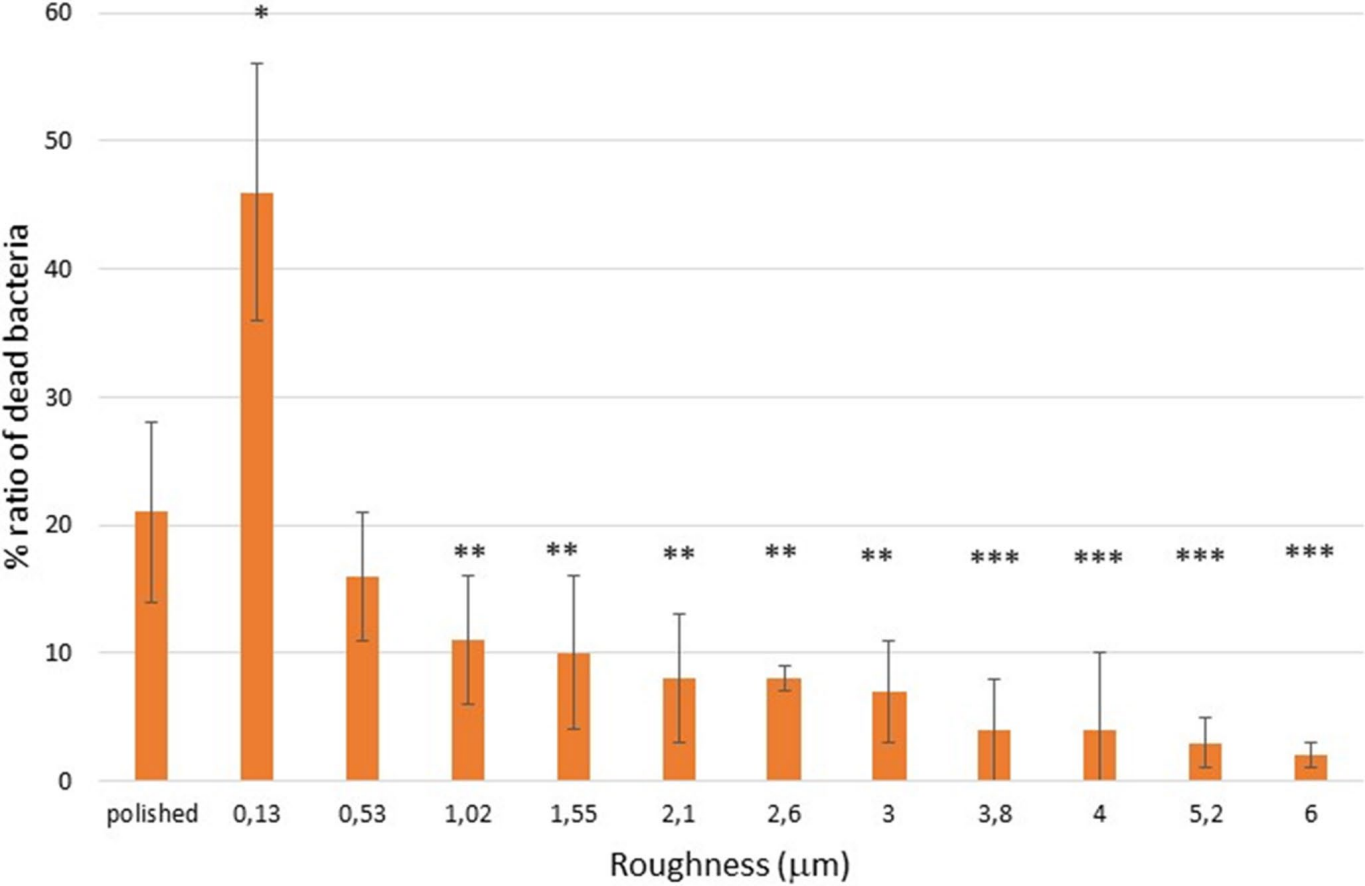
Percentage of the ratio of dead bacteria for the *Porphyromonas gingivalis* One asterisk is statistically different of two and three asterisks and two and three asterisks are different between them. The same meaning is for four asterisks. The p-values for each condition are shown in the Supplement. The bars are standard error of the mean. (*n* = 5 discs for each roughness studied)

**Fig. 7:**
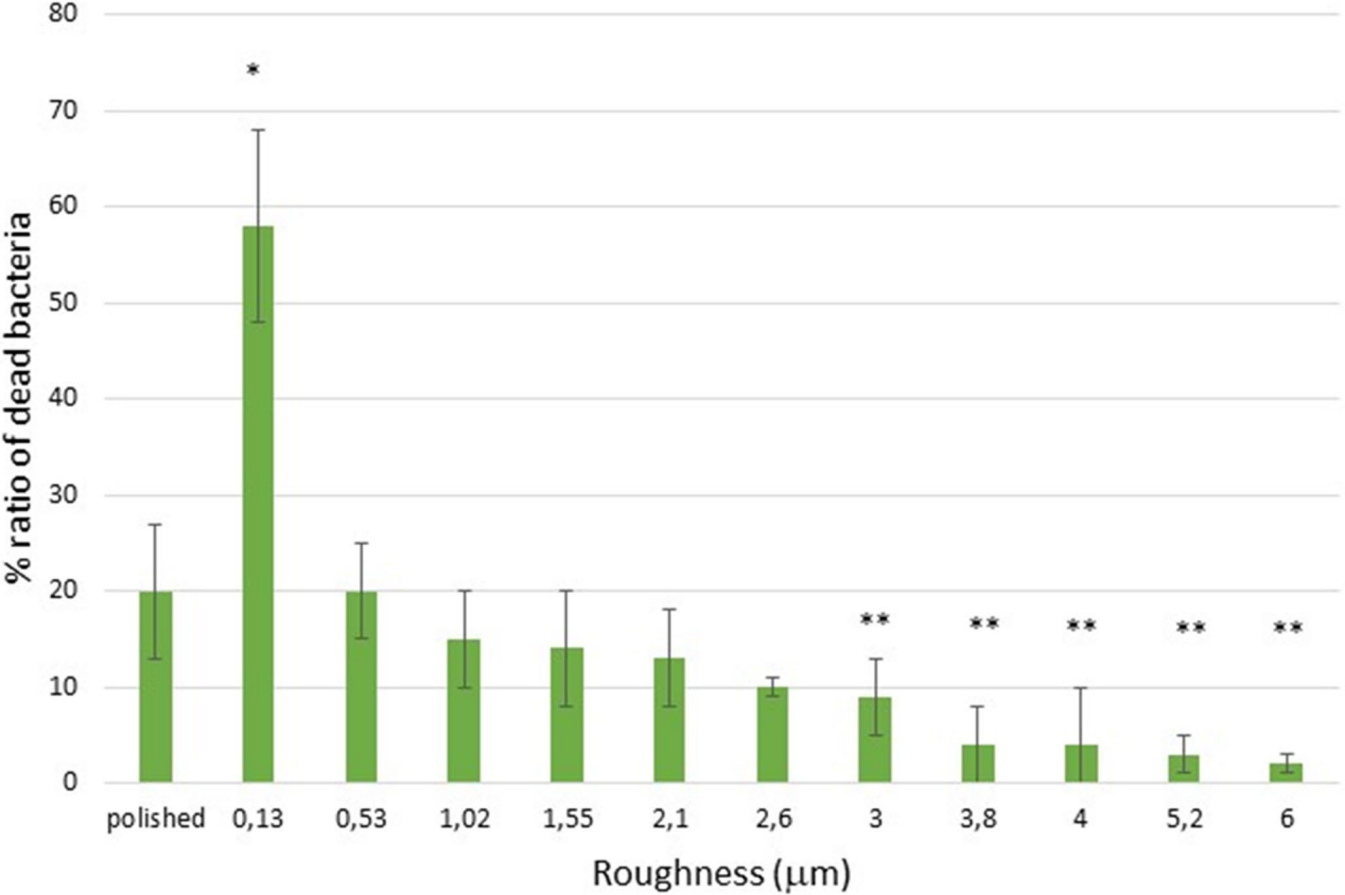
Percentage of the ratio of dead bacteria for the *Streptococcus sanguinis.* One asterisk is statistically different of two and three asterisks and two and three asterisks are different between them. The same meaning is for four asterisks. The p-values for each condition are shown in the Supplement. The bars are standard error of the mean. (*n* = 5 discs for each roughness studied)

When performing Live/Dead studies on each of the samples, the percentage of dead bacteria for each roughness could be calculated from the red and green colorations according to the equation cited above. This can be seen in Fig. 6 for the bacterial strain *Porphyromonas gingivalis* and in Fig. 7 for the *Streptococcus sanguinis*.

**Fig. 8:**
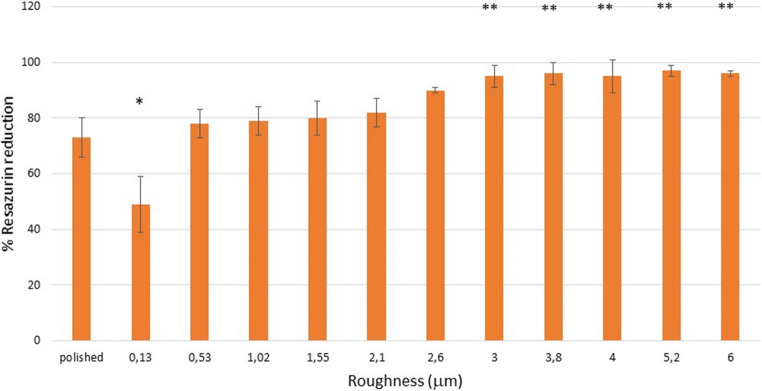
Percentage of the Resazurin reduction for the *Streptococcus sanguinis* One asterisk is statistically different of two asterisks. The p-values for each condition are shown in the Supplement. The bars are standard error of the mean. (*n* = 5 discs for each roughness studied

**Fig. 9:**
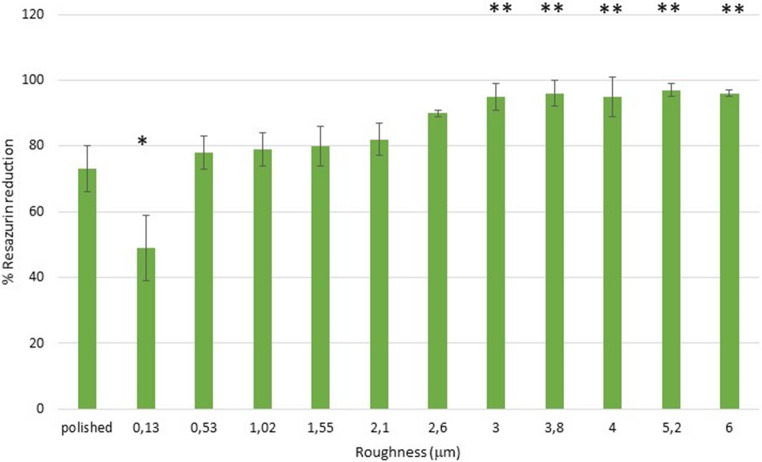
Percentage of the Resazurin reduction for the *Streptococcus sanguinis* One asterisk is statistically different of two asterisks. The p-values for each condition are shown in the Supplement. The bars are standard error of the mean. (n = 5 discs for each roughness studied

The results of the metabolic activity, determined by Resazurin reduction, of *Porphyromonas gingivalis* can be observed in Fig. 8 and for *Streptococcus sanguinis* in Fig. 9.

**Fig. 10:**
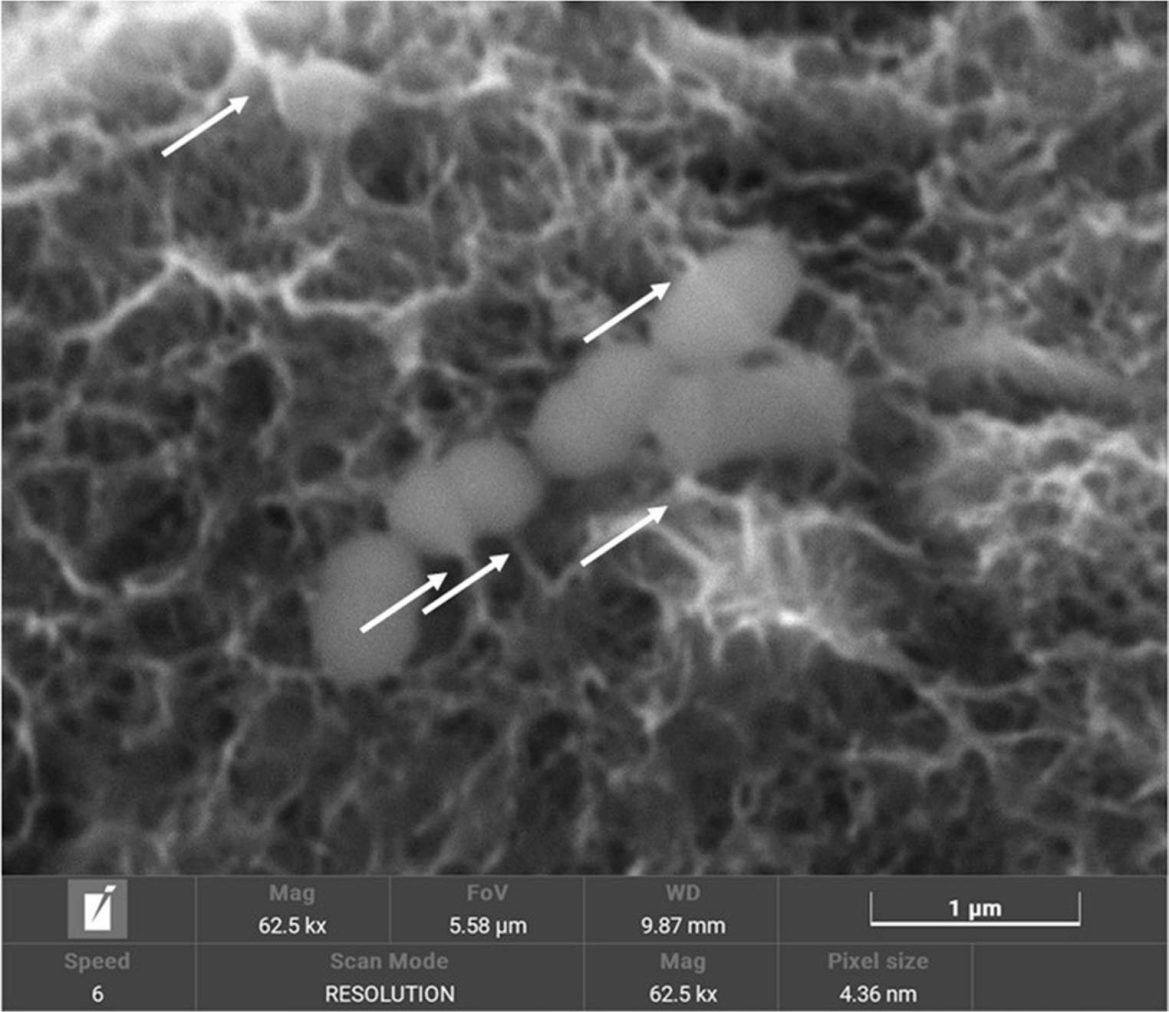
*Streptococcus sanguinis on* the titanium surface. The arrows indicate the interaction of the pillars with the bacteria

Using high resolution electron microscopy. observations were made at 62.500 magnifications where the interaction of the roughness with the bacteria could be resolved. as can be seen in Fig. 10. The peaks of the roughness act as spikes that pierce the membrane of the bacteria as they attempt to colonize the surface and therefore bacterial death. This fact is not seen in the cells since the size of the cells is much larger and their interaction does not harm them.

## Discussion

Peri-implant infections. primarily caused by biofilm formation on the implant surface (peri-implantitis). are the leading cause of implant failure. with a reported prevalence ranging from 1% to 47%. These infections trigger an immune-inflammatory response in the peri-implant tissues. ultimately resulting in tissue loss [31]. To address this issue. researchers have explored various strategies. including the development of implant surfaces designed to reduce bacterial adhesion and biofilm formation. Surface roughness is a critical factor influencing bacterial adherence; however. most studies have only compared a few commercially available surfaces with machined titanium. In contrast. our study investigates a wide range of rough surfaces. with roughness values between 0.1 μm and 6 μm. created by alumina shot blasting. We evaluated the adhesion capacity of two bacterial strains commonly involved in peri-implant biofilm formation across all these surfaces.

Firstly. we have been able to determine the versatility of the shot blasting treatment to achieve the desired roughness. This technique allows with the size of the abrasive particle. distance from the gun to the surface and the projection pressure to obtain roughness values to obtain the best compromise in the best osteoblastic activity and the worst microbiological activity [32, 33].

This study was carried out with alumina particles since they have an adequate abrasiveness compared to pure titanium. Titanium oxides or calcium phosphates do not have sufficient abrasiveness and do not cause significant roughness on titanium. Other abrasive material such as silicon carbide is not suitable either as it has been proven that in contact with physiological fluids it can cause a reaction with the formation of silicon oxide and carbon is susceptible to react with titanium. Some authors have shown that residual alumina on the surface of titanium favors osteoblastic activity since aluminum oxide has a negative polar component that favors the adsorption of fibronectin which is a precursor of osteoblastic migration to the surface [34]. In addition. these authors also suggest a certain antibacterial character of alumina due to the oxidizing character of alumina that can cause oxidation of bacteria [35].

From the results we could also appreciate that the roughness does not affect the contact angles. i.e. the wettability is not affected by the roughness (corrected with the Wenzel equation) but only by the nature of the material. However. when we compare the contact angles between polished and rough materials. there is a statistically significant difference of about 15º. This is due to the fact that the polished sample presents very low residual stress values with respect to the compressive residual stress of the rough materials. which for all cases is between − 120 and − 150 MPa (the negative sign means that the stress has compressive character) [36].

The effects of various implant surface decontamination techniques on implant surface roughness and wettability have recently been highlighted. Techniques such as laser. air polishing. ultraviolet (UV) light and cold atmospheric plasma used for decontamination present different degrees of modification of the roughness of the implant surface and different variations in wettability. and there is no correlation between both concepts in the different existing papers. as in our study [37]. The lack of correlation between surface roughness and wettability could be justified because bacteria can develop survival mechanisms such as hydrogen bonds. as hydrophobic bacteria adhere to hydrophilic surfaces. This is because bacteria are composed of 70% water. which allows non-specific binding to a hydrophilic surface [38, 39].

The influence of compressive residual stress has been shown to have a positive effect on the adhesion behavior. proliferation and osteoblastic differentiation due to the fact that having a greater hydrophilicity produces a greater protein adsorption and therefore cell adhesion is greater. especially that of the osteoblasts. if the titanium has been treated by shot blasting with alumina particles [39]. This in vitro behavior has been verified by Pereira et al. in vivo studies on New Zealand rabbits that after placing rough dental implants by shot blasting with residual stress and after eliminating the residual stress by heat treatment at 850 °C for 1 h. a BIC of approximately 8% higher for implants with residual stress could be verified [40].

The reason that for roughness of 0.13 mm there is a lower number of bacterial colonies is due to the fact that the topography causes spikes that the bacteria cannot adsorb on the surface or with great difficulty. This fact has been proven in chemical passivation treatments called Piranha. where the nanospikes can cause bacterial death (Fig. 9) [41]. The authors also comment that the cellular behavior of both osteoblastic and fibroblastic cells is not affected due to the much larger size of these cells with respect to the bacteria [41, 42].

The greater roughness causes an increase in bacterial colonies. which will cause significant increases in bacterial proliferation and biofilm creation. In our study it has been observed that the behavior of Gram + and Gram- bacteria. that play an important role in the creation of biofilms and cause peri-implantitis. are very similar.

The results of the Live/Dead tests and those of metabolic activity confirm the results obtained in bacterial colonization, where surfaces with a roughness of 0.13 micrometers have a higher ratio of dead bacteria and lower metabolic activity than polished surfaces with a roughness of 0.01 micrometers of Sa. Likewise, statistically significant differences can also be seen with roughnesses between 0.53 and 6 micrometers. The cause of the bactericidal activity of this surface can only be due to the spike-shaped topography of the surface, since there is no change in the chemical nature of the passivation layer, no antibiotic substances have been added, and the physical-chemical properties of the surfaces studied do not show statistically significant changes with *p* < 0.05.

With the results of the work by Romero et al. where they studied the influence of implant roughness on primary stability. and where the most appropriate values were when the Sa was greater than 1 μm and up to 4 μm]. and with the results obtained in this work with Gram + and Gram- bacteria involved in peri-implantitis. we can affirm that from a clinical point of view. it would not be appropriate to exceed 2 μm of roughness on the surface of the implants. Therefore. the optimum compromise values would be to obtain roughness be between 1 and 2 μm in order to have good osteoblastic and worse bacterial behavior [43, 44].

Several studies have attempted to relate surface roughness to the colonization of different bacterial strains. Bevilacqua et al. [45] investigated the amount of biofilm formed on different roughness of treated titanium. both in vivo and in vitro. They found that in vitro biofilm development was more strongly influenced by surface roughness than biofilm formation in vivo. They conclude that quantitative differences in biofilm formation measured in vitro using one or few bacterial species may not reliably predict colonization rates in vivo. Conserva et al. [46] reached similar conclusions in an in vivo study in which they evaluated plaque accumulation on three different commercial surfaces of varying roughness. They observed plaque formation on all surfaces. but with no significant difference between them. concluding that the level of roughness does not appear to be a critical factor for plaque accumulation in vivo.

Some researchers studied bacterial adhesion to different surface roughness. using a multispecies biofilm model. which more closely resembles the conditions in the oral cavity than models using isolated bacterial strains. So. Bermejo et al. [47] reported greater bacterial accumulation on moderately rough surfaces (Sa 1–2 μm). compared to minimally rough surfaces (Sa 0.5–1 μm) using a similar biofilm structure. From the same group of researchers. Bravo et al. [48] carried out an in vitro study comparing a turned surface (Sa 0.18 μm) with a moderately roughened one (Sa 1.53 μm). They found that significantly more biofilm developed on the moderately rough surface than on the smooth surface. Our studies support these results; moreover. by examining a larger number of rough surfaces. all prepared using the same method. we were able to generate a more detailed map of bacterial adhesion capacity according to surface roughness.

Recent studies have explored bacterial adhesion to surfaces with varying roughness. using both titanium and zirconia as substrates. Although at present titanium is still moderately the most widely used material in the manufacture of dental implants. there is more and more research and experience on the use of zirconia as a biocompatible material capable of competing with titanium in implantology. In this regard. Choi et al. [49] studied the adhesion of different strains on two different roughness. smooth and moderately rough. on both titanium and zirconium specimens. The zirconia surface retained less plaque. but the authors noted that bacterial growth was more dependent on the bacterial species than on the surface. The same conclusion was reached by Wassmann et al. [50] in a similar study. However. authors such as Roehling et al. [51] or Siddiqui et al. [52] do not find differences between. zirconia or titanium. but it is the roughness that has an influence. so that the greater the roughness. the greater the bacterial adherence. regardless of the material used. Further research is therefore needed on the adhesion and proliferation capacity of different oral bacteria on Zirconia implants. assessing the role of different levels of microroughness on the behavior of bacteria in relation to the titanium.

The originality of this work has consisted in the fact that the studies have been made for the same titanium grade 3 material from the same batch. shot blasting treatments have been made with the same abrasive with different sizes. The wettability. residual compressive stress caused by shot blasting and the amount of residual alumina remaining on the surface have been kept constant. so that only the roughness will affect the bacterial colonization behavior, ratio of the dead bacteria and the metabolic activity. It has been difficult to compare the results since most of the works use dental implants with different designs and different roughness. but the processes and other physicochemical characteristics that may affect the microbiological response are not described. In this case. a range of different roughness have been obtained only with the modification of the sizes of the alumina abrasive particles.

However. the work has some limitations since the number of bacteria in the study should be increased as well as the behavior of biofilms. In the same way. further studies should be carried out on the antibacterial mechanism in very small roughness since this behavior may depend on the type of bacteria. the tenacity of their membranes or the morphology and morphometry of the bacteria among others [53–58].

We have also seen that the amount of residual alumina in the shot blasting process is constant on the surface and also the literature comments that it has a slight antibacterial activity, so it would be interesting to carry out studies in the absence of these particles to determine their behavior for all roughness. In the same way. it would occur with the values of compressive residual stresses that have been shown to have a biological influence. However. despite these limitations in this work, it is shown that roughness favors the increase of colonies of the Gram + and Gram- bacteria studied and that very small roughness present fewer colonies than specular polished surfaces. These results may be of interest to dental implant and abutment manufacturers, as they could help guide the development of surface roughness that supports tissue growth while limiting bacterial colonization.

## Conclusion

The findings of this study demonstrate that shot blasting treatment with alumina of different particle sizes can generate a broad range of surface roughness values. When projection distance and abrasive pressure are kept constant, no statistically significant differences were observed in physicochemical properties of the titanium surface (wettability, compressive residual stress, and residual alumina). In all cases, blasted surfaces exhibited greater hydrophilicity and higher residual stress compared to polished titanium.

Bacterial colonization by *Porphyromonas gingivalis* and *Streptococcus sanguinis* increased with increasing roughness, with both Gram-positive and Gram-negative strains showing a similar trend. Colony formation rose gradually at lower roughness values and sharply beyond 3.0 μm for Gram-positive and 2.1 μm for Gram-negative bacteria. From a clinical perspective, roughness values exceeding 2.0 μm may be undesirable, as they promote excessive bacterial colonization. Optimal compromise values appear to lie between 1.0 and 2.0 μm, balancing favorable osteoblastic responses with reduced bacterial adhesion.

Surfaces with an approximate roughness of 0.13 μm showed even less bacterial colonization than the polished surface (0.01 μm), probably due to the penetration of the titanium abutments into the bacteria. This observation was supported by the increase in the proportion of dead bacteria, the reduction in bacterial metabolic activity, and high-resolution electron microscopy observation of these surfaces.

Within the limitations of this in vitro study, these results provide valuable insights for implant manufacturers. Optimizing surface roughness may enhance bone–implant integration while simultaneously reducing bacterial colonization, thereby contributing to the prevention of peri-implantitis.

## Supplementary Information

Below is the link to the electronic supplementary material.

Supplementary Material 1 (DOCX 31.6 KB)

## Data Availability

No datasets were generated or analysed during the current study.
