# Supplementary material for: Influence of alumina shot blasting induced roughness on bacterial adhesion to titanium
Source: Clin Oral Investig. 2025 Oct 11;29(11):497. doi: 10.1007/s00784-025-06580-2 (PMC12515120; doi:10.1007/s00784-025-06580-2)
Supplement: Supplementary file 1 — Supplementary Material 1 (DOCX 31.6 KB) [file 784_2025_6580_MOESM1_ESM.docx]

**Physico-chemical Properties.** P-vales of Contact Angle (CA), percentage of alumina debris (alumina) and compressive residual stress (CRS)

| Roughness Sa(μm) | p-CA | p-alumina | p-CRS |
| --- | --- | --- | --- |
| 0.01 vs 0.13 | 0.018 | 0.025 | 0.008 |
| 0.01 vs 0.53 | 0.020 | 0.028 | 0.009 |
| 0.01 vs 1.02 | 0.015 | 0.031 | 0.011 |
| 0.01 vs 1.55 | 0.014 | 0.029 | 0.012 |
| 0.01 vs 2.10 | 0.019 | 0.041 | 0.007 |
| 0.01 vs 2.60 | 0.015 | 0.013 | 0.009 |
| 0.01 vs 3.00 | 0.018 | 0.019 | 0.016 |
| 0.01 vs 3.80 | 0.021 | 0.025 | 0.018 |
| 0.01 vs 4.00 | 0.027 | 0.029 | 0.015 |
| 0.01 vs 5.20 | 0.015 | 0.035 | 0.018 |
| 0.01 vs 6.00 | 0.017 | 0.028 | 0.009 |
| 0.13 vs 0.53 | > 0.05 | > 0.05 | > 0.05 |
| 0.13 vs 1.02 | > 0.05 | > 0.05 | > 0.05 |
| 0.13 vs 1.55 | > 0.05 | > 0.05 | > 0.05 |
| 0.13 vs 2.10 | > 0.05 | > 0.05 | > 0.05 |
| 0.13 vs 2.60 | > 0.05 | > 0.05 | > 0.05 |
| 0.13 vs 3.00 | > 0.05 | > 0.05 | > 0.05 |
| 0.13 vs 3.80 | > 0.05 | > 0.05 | > 0.05 |
| 0.13 vs 4.00 | > 0.05 | > 0.05 | > 0.05 |
| 0.13 vs 5.20 | > 0.05 | > 0.05 | > 0.05 |
| 0.13 vs 6.00 | > 0.05 | > 0.05 | > 0.05 |
| 0.53 vs 1.02 | > 0.05 | > 0.05 | > 0.05 |
| 0.53 vs 1.55 | > 0.05 | > 0.05 | > 0.05 |
| 0.53 vs 2.10 | > 0.05 | > 0.05 | > 0.05 |
| 0.53 vs 2.60 | > 0.05 | > 0.05 | > 0.05 |
| 0.53 vs 3.00 | > 0.05 | > 0.05 | > 0.05 |
| 0.53 vs 3.80 | > 0.05 | > 0.05 | > 0.05 |
| 0.53 vs 4.00 | > 0.05 | > 0.05 | > 0.05 |
| 0.53 vs 5.20 | > 0.05 | > 0.05 | > 0.05 |
| 0.53 vs 6.00 | > 0.05 | > 0.05 | > 0.05 |
| 1.02 vs 1.55 | > 0.05 | > 0.05 | > 0.05 |
| 1.02 vs 2.10 | > 0.05 | > 0.05 | > 0.05 |
| 1.02 vs 2.60 | > 0.05 | > 0.05 | > 0.05 |
| 1.02 vs 3.00 | > 0.05 | > 0.05 | > 0.05 |
| 1.02 vs 3.80 | > 0.05 | > 0.05 | > 0.05 |
| 1.02 vs 4.00 | > 0.05 | > 0.05 | > 0.05 |
| 1.02 vs 5.20 | > 0.05 | > 0.05 | > 0.05 |
| 1.02 vs 6.00 | > 0.05 | > 0.05 | > 0.05 |
| 1.55 vs 2.10 | > 0.05 | > 0.05 | > 0.05 |
| 1.55 vs 2.60 | > 0.05 | > 0.05 | > 0.05 |
| 1.55 vs 3.00 | > 0.05 | > 0.05 | > 0.05 |
| 1.55 vs 3.80 | > 0.05 | > 0.05 | > 0.05 |
| 1.55 vs 4.00 | > 0.05 | > 0.05 | > 0.05 |
| 1.55 vs 5.20 | > 0.05 | > 0.05 | > 0.05 |
| 1.55 vs 6.00 | > 0.05 | > 0.05 | > 0.05 |
| 2.10 vs 2.60 | > 0.05 | > 0.05 | > 0.05 |
| 2.10 vs 3.00 | > 0.05 | > 0.05 | > 0.05 |
| 2.10 vs 3.80 | > 0.05 | > 0.05 | > 0.05 |
| 2.10 vs 4.00 | > 0.05 | > 0.05 | > 0.05 |
| 2.10 vs 5.20 | > 0.05 | > 0.05 | > 0.05 |
| 2.10 vs 6.00 | > 0.05 | > 0.05 | > 0.05 |
| 2.60 vs 3.00 | > 0.05 | > 0.05 | > 0.05 |
| 2.60 vs 3.80 | > 0.05 | > 0.05 | > 0.05 |
| 2.60 vs 4.00 | > 0.05 | > 0.05 | > 0.05 |
| 2.60 vs 5.20 | > 0.05 | > 0.05 | > 0.05 |
| 2.60 vs 6.00 | > 0.05 | > 0.05 | > 0.05 |
| 3.00 vs 3.80 | > 0.05 | > 0.05 | > 0.05 |
| 3.00 vs 4.00 | > 0.05 | > 0.05 | > 0.05 |
| 3.00 vs 5.20 | > 0.05 | > 0.05 | > 0.05 |
| 3.00 vs 6.00 | > 0.05 | > 0.05 | > 0.05 |
| 3.80 vs 4.00 | > 0.05 | > 0.05 | > 0.05 |
| 3.80 vs 5.20 | > 0.05 | > 0.05 | > 0.05 |
| 3.80 vs 6.00 | > 0.05 | > 0.05 | > 0.05 |
| 4.00 vs 5.20 | > 0.05 | > 0.05 | > 0.05 |
| 4.00 vs 6.00 | > 0.05 | > 0.05 | > 0.05 |
| 5.20 vs 6.00 | > 0.05 | > 0.05 | > 0.05 |

***Porphyromonas gingivalis.* P-vales for each roughness for the Colonies Formation units, percentage of ratio of dead bacteria and percentage for metabolic activity by resazurin reduction**

| Roughness  Sa(μm) | CFU/mm^2^ | % Ratio dead | % Res Red |
| --- | --- | --- | --- |
| 0.01 vs 0.13 | 0.038 | 0.022 | > 0.05 |
| 0.01 vs 0.53 | > 0.05 | > 0.05 | 0.034 |
| 0.01 vs 1.02 | > 0.05 | 0.031 | > 0.05 |
| 0.01 vs 1.55 | > 0.05 | 0.029 | > 0.05 |
| 0.01 vs 2.10 | > 0.05 | 0.041 | > 0.05 |
| 0.01 vs 2.60 | 0.033 | 0.013 | > 0.05 |
| 0.01 vs 3.00 | 0.035 | 0.019 | > 0.05 |
| 0.01 vs 3.80 | 0.034 | 0.025 | > 0.05 |
| 0.01 vs 4.00 | 0.023 | 0.029 | > 0.05 |
| 0.01 vs 5.20 | 0.041 | 0.035 | > 0.05 |
| 0.01 vs 6.00 | 0.037 | 0.031 | > 0.05 |
| 0.13 vs 0.53 | 0.014 | 0.012 | 0.022 |
| 0.13 vs 1.02 | 0.025 | 0.023 | 0.025 |
| 0.13 vs 1.55 | 0.035 | 0.025 | 0.012 |
| 0.13 vs 2.10 | 0.041 | 0.042 | 0.036 |
| 0.13 vs 2.60 | 0.042 | 0.036 | 0.028 |
| 0.13 vs 3.00 | 0.043 | 0.036 | 0.029 |
| 0.13 vs 3.80 | 0.032 | 0.025 | 0.029 |
| 0.13 vs 4.00 | 0.025 | 0.014 | 0.027 |
| 0.13 vs 5.20 | 0.014 | 0.035 | 0.036 |
| 0.13 vs 6.00 | 0.019 | 0.038 | 0.009 |
| 0.53 vs 1.02 | > 0.05 | 0.019 | > 0.05 |
| 0.53 vs 1.55 | > 0.05 | 0.025 | > 0.05 |
| 0.53 vs 2.10 | > 0.05 | 0.029 | > 0.05 |
| 0.53 vs 2.60 | 0.025 | 0.035 | > 0.05 |
| 0.53 vs 3.00 | 0.028 | 0.031 | 0.028 |
| 0.53 vs 3.80 | 0.036 | 0.012 | 0.036 |
| 0.53 vs 4.00 | 0.041 | 0.025 | 0.041 |
| 0.53 vs 5.20 | 0.026 | 0.045 | 0.025 |
| 0.53 vs 6.00 | 0.033 | 0.041 | 0.036 |
| 1.02 vs 1.55 | > 0.05 | > 0.05 | > 0.05 |
| 1.02 vs 2.10 | > 0.05 | > 0.05 | > 0.05 |
| 1.02 vs 2.60 | 0.045 | > 0.05 | > 0.05 |
| 1.02 vs 3.00 | 0.044 | > 0.05 | 0.028 |
| 1.02 vs 3.80 | 0.040 | > 0.05 | 0.029 |
| 1.02 vs 4.00 | 0.039 | > 0.05 | 0.035 |
| 1.02 vs 5.20 | 0.025 | > 0.05 | 0.033 |
| 1.02 vs 6.00 | 0.026 | > 0.05 | 0.022 |
| 1.55 vs 2.10 | > 0.05 | > 0.05 | > 0.05 |
| 1.55 vs 2.60 | 0.031 | > 0.05 | > 0.05 |
| 1.55 vs 3.00 | 0.029 | > 0.05 | 0.028 |
| 1.55 vs 3.80 | 0.041 | 0.025 | 0.045 |
| 1.55 vs 4.00 | 0.013 | 0.028 | 0.036 |
| 1.55 vs 5.20 | 0.019 | 0.090 | 0.028 |
| 1.55 vs 6.00 | 0.025 | 0.035 | 0.027 |
| 2.10 vs 2.60 | 0.035 | > 0.05 | > 0.05 |
| 2.10 vs 3.00 | 0.036 | > 0.05 | 0.035 |
| 2.10 vs 3.80 | 0.041 | 0.033 | 0.028 |
| 2.10 vs 4.00 | 0.048 | 0.041 | 0.036 |
| 2.10 vs 5.20 | 0.044 | 0.036 | 0.036 |
| 2.10 vs 6.00 | 0.045 | 0.041 | 0.041 |
| 2.60 vs 3.00 | > 0.05 | > 0.05 | 0.045 |
| 2.60 vs 3.80 | > 0.05 | 0.041 | 0.045 |
| 2.60 vs 4.00 | 0.041 | 0.041 | 0.044 |
| 2.60 vs 5.20 | 0.026 | 0.025 | 0.025 |
| 2.60 vs 6.00 | 0.033 | 0.040 | 0.036 |
| 3.00 vs 3.80 | > 0.05 | 0.045 | > 0.05 |
| 3.00 vs 4.00 | 0.045 | 0.048 | > 0.05 |
| 3.00 vs 5.20 | 0.035 | 0.047 | > 0.05 |
| 3.00 vs 6.00 | 0.033 | > 0.05 | > 0.05 |
| 3.80 vs 4.00 | 0.025 | > 0.05 | > 0.05 |
| 3.80 vs 5.20 | 0.036 | > 0.05 | > 0.05 |
| 3.80 vs 6.00 | 0.047 | > 0.05 | > 0.05 |
| 4.00 vs 5.20 | > 0.05 | > 0.05 | > 0.05 |
| 4.00 vs 6.00 | > 0.05 | > 0.05 | > 0.05 |
| 5.20 vs 6.00 | > 0.05 | > 0.05 | > 0.05 |

***Streptococcus sanguinis.* P-vales for each roughness for the Colonies Formation units, percentage of ratio of dead bacteria and percentage for metabolic activity by resazurin reduction**

| Roughness  Sa(μm) | CFU/mm^2^ | % Ratio dead | % Res Red |
| --- | --- | --- | --- |
| 0.01 vs 0.13 | 0.028 | 0.028 | 0.039 |
| 0.01 vs 0.53 | 0.029 | > 0.05 | > 0.05 |
| 0.01 vs 1.02 | 0.029 | > 0.05 | > 0.05 |
| 0.01 vs 1.55 | 0.027 | > 0.05 | > 0.05 |
| 0.01 vs 2.10 | 0.023 | > 0.05 | > 0.05 |
| 0.01 vs 2.60 | 0.025 | > 0.05 | > 0.05 |
| 0.01 vs 3.00 | 0.042 | 0.025 | 0.033 |
| 0.01 vs 3.80 | 0.036 | 0.028 | 0.025 |
| 0.01 vs 4.00 | 0.036 | 0.047 | 0.014 |
| 0.01 vs 5.20 | 0.025 | 0.035 | 0.049 |
| 0.01 vs 6.00 | 0.036 | 0.022 | 0.040 |
| 0.13 vs 0.53 | 0.035 | 0.025 | 0.044 |
| 0.13 vs 1.02 | 0.028 | 0.012 | 0.046 |
| 0.13 vs 1.55 | 0.029 | 0.036 | 0.038 |
| 0.13 vs 2.10 | 0.035 | 0.028 | 0.032 |
| 0.13 vs 2.60 | 0.033 | 0.029 | 0.044 |
| 0.13 vs 3.00 | 0.028 | 0.029 | 0.032 |
| 0.13 vs 3.80 | 0.029 | 0.027 | 0.036 |
| 0.13 vs 4.00 | 0.035 | 0.036 | 0.021 |
| 0.13 vs 5.20 | 0.033 | 0.019 | 0.012 |
| 0.13 vs 6.00 | 0.028 | 0.014 | 0.009 |
| 0.53 vs 1.02 | > 0.05 | > 0.05 | > 0.05 |
| 0.53 vs 1.55 | > 0.05 | > 0.05 | > 0.05 |
| 0.53 vs 2.10 | > 0.05 | > 0.05 | > 0.05 |
| 0.53 vs 2.60 | > 0.05 | > 0.05 | > 0.05 |
| 0.53 vs 3.00 | > 0.05 | 0.023 | 0.012 |
| 0.53 vs 3.80 | 0.045 | 0.048 | 0.022 |
| 0.53 vs 4.00 | 0.035 | 0.047 | 0.028 |
| 0.53 vs 5.20 | 0.033 | 0.012 | 0.038 |
| 0.53 vs 6.00 | 0.025 | 0.025 | 0.047 |
| 1.02 vs 1.55 | 0.036 | > 0.05 | > 0.05 |
| 1.02 vs 2.10 | 0.041 | > 0.05 | > 0.05 |
| 1.02 vs 2.60 | 0.045 | > 0.05 | > 0.05 |
| 1.02 vs 3.00 | > 0.05 | 0.048 | 0.013 |
| 1.02 vs 3.80 | 0.025 | 0.025 | 0.014 |
| 1.02 vs 4.00 | 0.026 | 0.022 | 0.028 |
| 1.02 vs 5.20 | 0.037 | 0.024 | 0.027 |
| 1.02 vs 6.00 | 0.033 | 0.027 | 0.028 |
| 1.55 vs 2.10 | > 0.05 | > 0.05 | > 0.05 |
| 1.55 vs 2.60 | > 0.05 | > 0.05 | > 0.05 |
| 1.55 vs 3.00 | > 0.05 | 0.036 | 0.021 |
| 1.55 vs 3.80 | 0.032 | 0.033 | 0.020 |
| 1.55 vs 4.00 | 0.039 | 0.023 | 0.010 |
| 1.55 vs 5.20 | 0.038 | 0.045 | 0.032 |
| 1.55 vs 6.00 | 0.035 | 0.032 | 0.030 |
| 2.10 vs 2.60 | > 0.05 | 0.025 | > 0.05 |
| 2.10 vs 3.00 | > 0.05 | 0.025 | 0.040 |
| 2.10 vs 3.80 | 0.033 | 0.026 | 0.041 |
| 2.10 vs 4.00 | 0.028 | 0.033 | 0.040 |
| 2.10 vs 5.20 | 0.018 | 0.018 | 0.039 |
| 2.10 vs 6.00 | 0.044 | 0.036 | 0.034 |
| 2.60 vs 3.00 | > 0.05 | 0.025 | 0.033 |
| 2.60 vs 3.80 | 0.044 | 0.043 | 0.022 |
| 2.60 vs 4.00 | 0.046 | 0.044 | 0.023 |
| 2.60 vs 5.20 | 0.038 | 0.022 | 0.022 |
| 2.60 vs 6.00 | 0.032 | 0.043 | 0.031 |
| 3.00 vs 3.80 | 0.044 | > 0.05 | > 0.05 |
| 3.00 vs 4.00 | 0.043 | > 0.05 | > 0.05 |
| 3.00 vs 5.20 | 0.040 | > 0.05 | > 0.05 |
| 3.00 vs 6.00 | 0.041 | > 0.05 | > 0.05 |
| 3.80 vs 4.00 | > 0.05 | > 0.05 | > 0.05 |
| 3.80 vs 5.20 | > 0.05 | > 0.05 | > 0.05 |
| 3.80 vs 6.00 | > 0.05 | > 0.05 | > 0.05 |
| 4.00 vs 5.20 | > 0.05 | > 0.05 | > 0.05 |
| 4.00 vs 6.00 | > 0.05 | > 0.05 | > 0.05 |
| 5.20 vs 6.00 | > 0.05 | > 0.05 | > 0.05 |
